# Small Peptides Able to Suppress Prostaglandin E_2_ Generation in Renal Mesangial Cells

**DOI:** 10.3390/molecules23010158

**Published:** 2018-01-13

**Authors:** Sofia Vasilakaki, Oleksandr Pastukhov, Thomas Mavromoustakos, Andrea Huwiler, George Kokotos

**Affiliations:** 1 Laboratory of Organic Chemistry, Department of Chemistry, National and Kapodistrian University of Athens, Panepistimiopolis, Athens 15771, Greece; svasilak@chem.uoa.gr (S.V.); tmavrom@chem.uoa.gr (T.M.); 2 Institute of Pharmacology, University of Bern, Bern 3010, Switzerland; oleksandr.pastukhov@kispi.uzh.ch (O.P.); huwiler@pki.unibe.ch (A.H.)

**Keywords:** inhibitors, mesangial cells, peptides, prostaglandin E_2_, secreted phospholipase A_2_

## Abstract

Peptide drug discovery may play a key role in the identification of novel medicinal agents. Here, we present the development of novel small peptides able to suppress the production of PGE_2_ in mesangial cells. The new compounds were generated by structural alterations applied on GK115, a novel inhibitor of secreted phospholipase A_2_, which has been previously shown to reduce PGE_2_ synthesis in rat renal mesangial cells. Among the synthesized compounds, the tripeptide derivative **11** exhibited a nice dose-dependent suppression of PGE_2_ production, similar to that observed for GK115.

## 1. Introduction

Prostaglandin E_2_ (PGE_2_) is one of the most important lipid mediators [1] playing a pivotal role in inflammatory diseases [2] including glomerulonephritis. Glomerulonephritis is initiated by the invasion of activated immune cells into the renal glomerulus and the release of pro-inflammatory cytokines such as TNFα and interleukin (IL) 1β to activate the local resident mesangial cells [3,4]. This activation includes three main responses, i.e., increased proliferation, increased matrix production, and increased inflammatory mediator production, which are all hallmarks of glomerulonephritis, finally resulting in glomerulosclerosis and renal failure [4]. Due to this central involvement of mesangial cells in the pathogenesis of chronic inflammatory kidney diseases, they serve as a useful cellular model system to test for new therapeutic compounds to treat such diseases.

The biosynthesis of PGE_2_ is initiated by the action of phospholipases A_2_ (PLA_2_), which cleave the *sn*-2 ester bond of membrane glycerophospholipids to release arachidonic acid [5,6]. Then, arachidonic acid is converted to PGH_2_ and the terminal step of PGE_2_ generation is catalyzed by microsomal prostaglandin synthase-1 [7]. Four types of PLA_2_ [6] are expressed at mesangial cells: cytosolic (cPLA_2_) GIVA, calcium-independent (iPLA_2_) GVIA and secreted (sPLA_2_) GIIA and GV [8,9,10]. It has been demonstrated that the progression of glomerular inflammatory processes is a result of a network of interactions between different PLA_2_s [9] and a cross-talk between cPLA_2_ and sPLA_2_ has been reported in hydrogen peroxide-induced arachidonic acid release in murine mesangial cells [11]. In addition, sPLA_2_ GIIA seems to have multiple roles in mesangial cells inducing the production of pro-inflammatory mediators [12].

**Figure 1:**
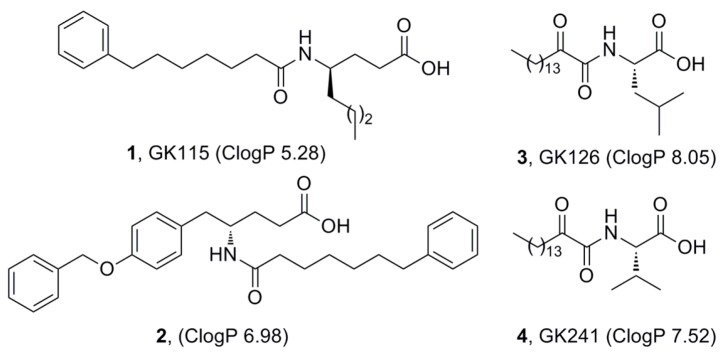
Inhibitors of secreted PLA_2_ able to suppress the formation of PGE_2_ in mesangial cells.

We have recently demonstrated that inhibitors of sPLA_2_ suppress the release of PGE_2_ formation in renal mesangial cells [13]. Four such inhibitors presented an interesting effect on the suppression of PGE_2_ release and their structures are shown in Figure 1. Inhibitors **1** (GK115) [14] and **2** [15] are amides based on the non-natural amino acids γ-norleucine and γ-tyrosine, respectively, while inhibitors **3** (GK126) [16] and **4** (GK241) [17] are 2-oxoamides based on the natural amino acids leucine and valine, respectively. As depicted in Figure 1, all of them are characterized by high lipophilicity (ClogP value higher than 5), an unfavorable property according to Lipinski’s rule of 5 [18]. The ClogP values of the inhibitors were calculated using ChemOffice Ultra 11.00.

**Figure 2:**
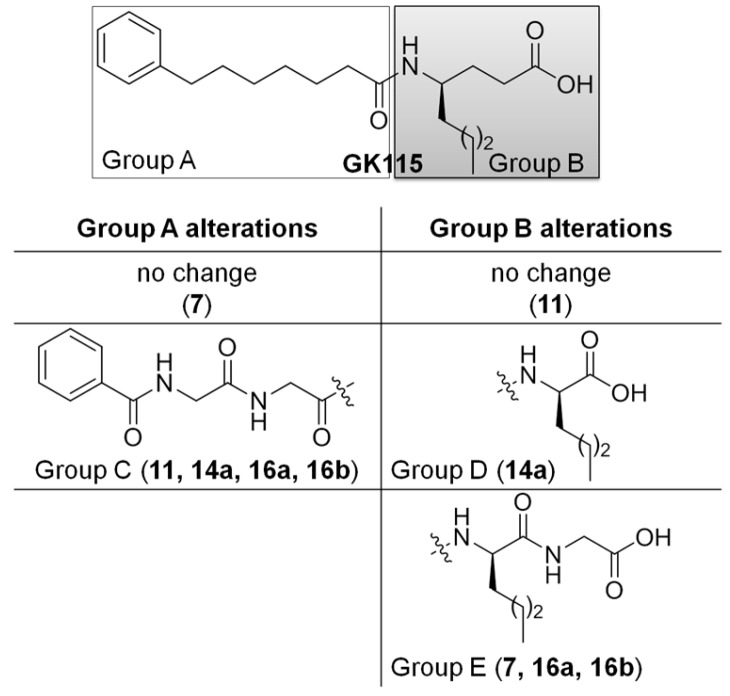
The design of new inhibitors was based on alterations of Groups A and B of GK115 structure. The table describes the alterations of Groups A and B and the corresponded generated compounds in brackets.

Over the last years, peptide drug discovery has experienced a revival of interest, as the pharmaceutical industry has come to appreciate the role that peptides may play in addressing unmet medical needs and how peptides can be an excellent complement to small molecule medicinal agents [19]. In an effort to identify novel compounds able to suppress the formation of PGE_2_, in the current work we explored the possibility of small conjugated peptides to inhibit the release of PGE_2_ at a cellular level. Herein, we report the structure-based design and synthesis of several small peptides derivatives (Figure 2), whose structures are inspired by the structures of sPLA_2_ inhibitors described above, and the evaluation of their ability to suppress PGE_2_ formation.

## 2. Results

### 2.1. Synthesis

Inspired by the structure of inhibitor GK115 and considering it as a lead compound, we designed derivatives replacing the γ-norleucine residue by α-norleucine or a norleucine based dipeptide. In addition, and in an effort to decrease the lipophilicity of the compounds, we replaced the acyl chain of GK115 (Group A, Figure 2) by a chain based on the benzoyl derivative of the peptide glycyl-glycine (Group C, Figure 2).

**Scheme 1:**
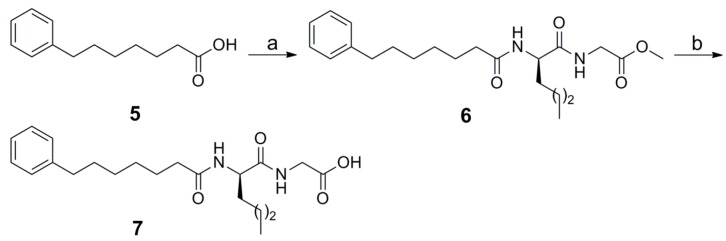
*Reagents and conditions*: a. H-Nle-Gly-OMe, WSCI·HCl, HOBt, Et_3_N, CH_2_Cl_2_; b. 1 N aq NaOH, MeOH.

Compound **7** was synthesized by coupling of 7-phenyl-heptanoic acid **5** with the dipeptide norleucyl-glycine using 1-(3-dimethylaminopropyl)-3-ethyl carbodiimide (WSCI) as a condensing agent in the presence of 1-hydroxybenzotriazole (HOBt), followed by alkaline hydrolysis (Scheme 1).

**Scheme 2:**
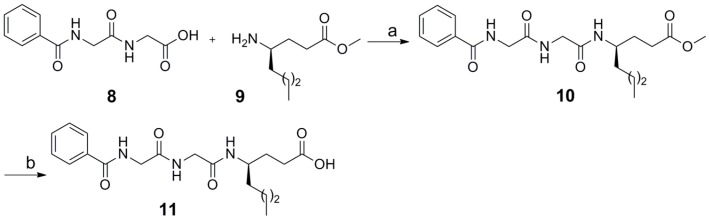
*Reagents and conditions*: a. WSCI·HCl, HOBt, Et_3_N, CH_2_Cl_2_; b. 1 N aq NaOH, MeOH.

Compound **11**, where the acyl chain of GK115 was replaced by the benzoyl derivative of the dipeptide glycyl-glycine, was synthesized by coupling of benzoyl-glycyl-glycine **8** with methyl γ-norleucinate **9**, as depicted in Scheme 2.

**Scheme 3:**
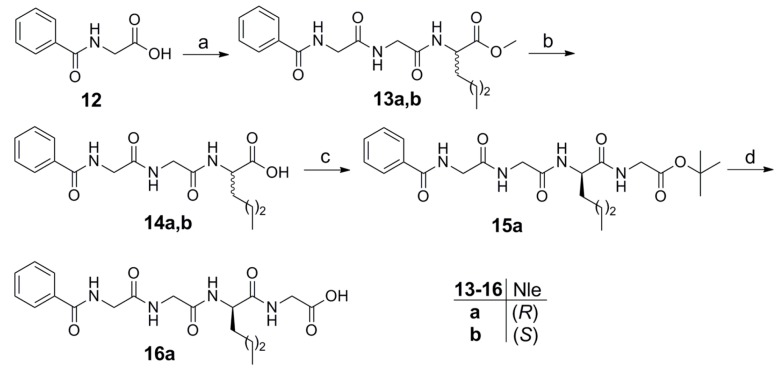
*Reagents and conditions*: a. H-Gly-Nle-OMe, WSCI·HCl, HOBt, Et_3_N, CH_2_Cl_2_; b. 1 N aq NaOH, MeOH; c. H-Gly-O*t*Bu, WSCI·HCl, HOBt, Et_3_N, CH_2_Cl_2_; d. CF_3_COOH, CH_2_Cl_2_.

To synthesize derivatives based on either α-norleucine or the dipeptide norleucyl-glycine, benzoyl-glycine **12** was coupled with methyl norleucyl-glycinate (Scheme 3). After alkaline hydrolysis, compounds **14a** and **14b** based on (*R*)- and (*S*)-norleucine, respectively, were obtained. Coupling of **14a** with *tert*-butyl glycinate led to compound **16a** after acidic hydrolysis.

### 2.2. Suppression of PGE_2_ Release in Mesangial Cells

Cultures of rat renal mesangial cells were stimulated for 24 h with IL-1β plus forskolin (Fk) to trigger a huge increase of PGE_2_ synthesis, as previously described [20]. Cells were treated in the absence or presence of three different concentrations (1 μM, 3 μM and 10 μM) of the various synthetic compounds. Supernatants were collected and subjected to PGE_2_-ELISA to quantify PGE_2_ released from the cells.

**Figure 3:**
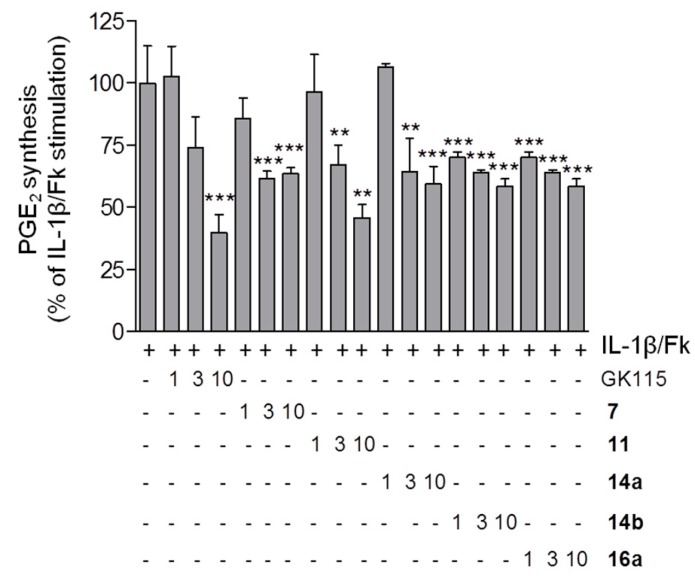
Effect of compounds **7**, **11**, **14a**, **14b** and **16a** on IL-1β/Fk-stimulated PGE_2_ formation in mesangial cells. Data are presented as % of maximal IL-1β/Fk stimulation and are means ± S.D. (*n* = 3). ** *p* < 0.01, *** *p* < 0.001 considered statistical significant when compared to the IL-1β/Fk samples. Basal PGE_2_ concentration was at 7.9 ± 5.3 pg/100 μL supernatant; the maximal IL-1β/Fk-stimulated PGE_2_ concentration was at 685 ± 81 pg/100 μL supernatant.

The results of the suppression of PGE_2_ release in mesangial cells caused by the new peptides derivatives are shown in Figure 3. In the absence of IL-1β plus Fk, none of the compounds, tested at the highest concentration of 10 μM, changed the basal PGE_2_ synthesis significantly (data not shown).

Most of the peptides derivatives tested were able to reduce the release of PGE_2_. The replacement of γ-norleucine by the dipeptide Nle-Gly (compound **7**) did not cause any remarkable difference in activity. The tripeptide derivative **11** exhibited a nice dose-dependent curve similar to that observed for GK115. The replacement of the hydrocarbon acyl chain of GK115 by the dipeptide glycyl-glycine chain resulted in a considerable decrease of the lipophilicity, dropping the ClogP value from 5.28 to 1.73. It should be noticed that this derivative seems efficiently protected by the action of both aminopeptidases and carboxypeptidases. Peptides based on γ-amino acids are resistant to carboxypeptidases [21], while the benzoyl protecting group offers resistance to aminopeptidases. Tripeptides **14a** and **14b** and tetrapeptide **16a** derivatives exhibited similar ability to suppress PGE_2_ formation irrespectively of either the stereochemistry of norleucine (*R* in **14a** and *S* in **14b**) or the size of the peptide (tripeptide versus tetrapeptide). Peptide incorporation of segments in **13a**, **14b** and **16a** resulted in reduced lipophilicities with ClogP values of 1.51, 1.51 and 0.94, respectively. These results confirm our hypothesis that we could improve compounds’ lipophilicity, while preserve their potency to suppress PGE_2_ release.

### 2.3. Docking Studies

Since we have shown that inhibition of sPLA_2_ is a possible path via which our sPLA_2_ inhibitors (GK115, GK126 and GK241) evoke the suppression of PGE_2_ release [13], we performed docking calculations for the binding pose prediction of compounds GK115 and **11** in the active site of the enzyme.

Sybyl 8.0 [22] by TRIPOS was used for the design and the energy minimization of the structures. The carboxyl group was considered deprotonated and the Powell algorithm was used for the energy minimization. The crystal structure of sPLA_2_ GIIA was retrieved from the Brookhaven Protein Databank (code: 1KQU) and used for the docking calculations. The protein’s missing loops were added with the Prime module of Schrödinger Suite [23]. The molecules were placed in the active site of the enzyme manually in Chimera [24] and docked in AutoDock Vina [25].

**Figure 4:**
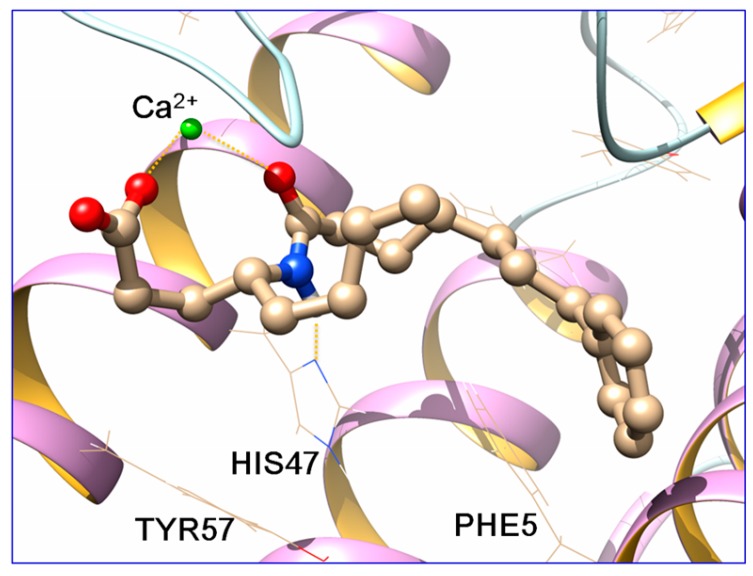
Binding pose prediction of GK115 in the active site of sPLA_2_ GIIA (red: oxygen atoms, blue: nitrogen atoms, green: calcium atom).

**Figure 5:**
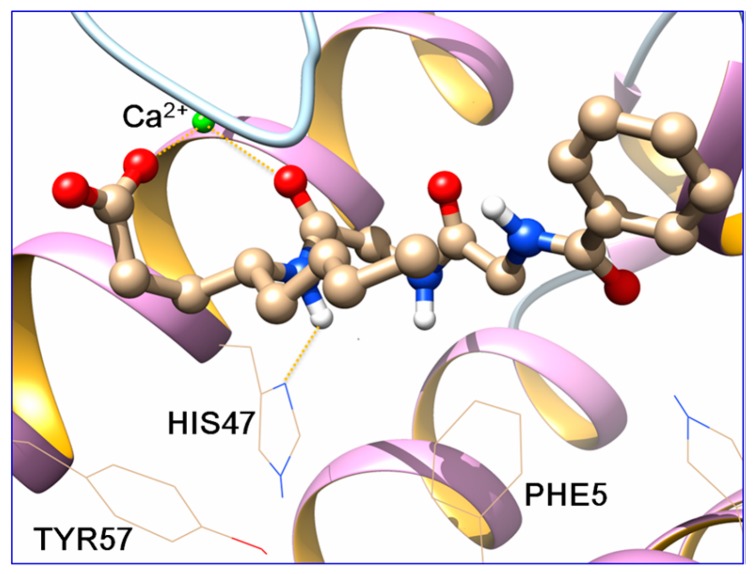
Binding pose prediction of **11** in the active site of sPLA_2_ GIIA (red: oxygen atoms, blue: nitrogen atoms, green: calcium atom).

By visual inspection of the docking results, docking poses which reproduced the known interactions between the co-crystallized ligands and the enzyme, were chosen and subjected to molecular dynamics (MD) simulations with AMBER14 [26], following a standard procedure [17]. MD simulations (100 ns) were run and the top cluster of each simulation is shown in Figure 4 and Figure 5. For the clustering, the hieragglo algorithm was used and the *averagelinkage* option in *cpptraj* [27] was defined using the Leu2, Phe5, His6, Ile9, Ala17-Gly32, Cys44, His47, Asp48, Lys62, Asp91 and Phe98 amino acids.

**Figure 6:**
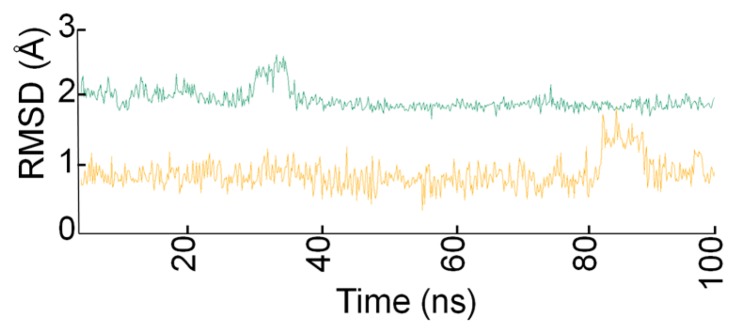
Root-mean-square deviation (RMSD) values of inhibitors **11** (green) and GK115 (orange) in the active site of sPLA_2_ GIIA during the MD simulation time.

The carboxyl group of both molecules and the carbonyl group of the Nle-Gly peptide bond of **11** and the amide group of GK115 chelate the Ca^2+^ ion throughout the simulation time. Additionally, a hydrogen bond is formed between His47 and the -NH group of the peptide bond of **11** and the amide group of GK115, respectively. These interactions are present in the crystallized structures of sPLA_2_ GIIA with the native ligands [15]. Both inhibitors adopt similar conformation in the active site and they preserve it, with small fluctuations, during the MD time (Figure 6).

This further contributes to the persistence of the interactions between the molecules and the residues of the active site. MM-PBSA [28] method was used for the estimation of the energetic terms of both systems. The Δ*H* value for inhibitor GK115 is −47.16 kcal/mol with a standard deviation error (SDE) 5.16 kcal/mol and for compound **11** is −38.22 kcal/mol with SDE of 6.26 kcal/mol. Since GK115 is a potent inhibitor of sPLA_2_ GIIA, both the key interactions and the Δ*H* values calculated by the MD trajectories are a good indicator of a possible inhibition of sPLA_2_ GIIA by **11** and biological assays will be performed in a future study to investigate this possibility.

## 3. Discussion

Our studies led to the identification of a number of small peptides derivatives that present a significant suppression of PGE_2_ release in renal mesangial cells. Among them, benzoyl-glycyl-glycyl-γ-(*R*)-norleucine (**11**) showed an interesting effect on the suppression of PGE_2_ formation. This tripeptide derivative has a significant lower lipophilicity in comparison to the lead compound GK115 without loss of the ability to suppress PGE_2_ release at a cellular level. Thus, new agents that can serve as leads to reduce pathological PGE_2_ production, as seen in chronic inflammatory kidney diseases such as glomerulonephritis, have been identified.

## 4. Materials and Methods

### 4.1. Chemistry

Merck Silica Gel 60 (70–230 or 230–400 mesh) (Merck Co., Darmstadt, Germany) was used for the chromatographic purification of products and Silica Gel 60 F254 (Merck Co., Darmstadt, Germany) aluminum plates for the thin-layer chromatography (TLC). UV light and/or phosphomolybdic acid in EtOH was employed for visualizing shpots. A Büchi 530 apparatus (Buchi, Flawil, Switzerland) was used to estimate melting points and they were uncorrected. ^1^H and ^13^C–NMR spectra were recorded on Varian Mercury (Varian Inc., Palo Alto, CA, USA) at 200 MHz and 50 MHz respectively. Samples were diluted in CDCl_3_, CD_3_OD, DMSO or D_2_O. Chemical shifts are given in ppm, and coupling constants (*J*) in Hz. Peak multiplicities are typified as: s, singlet, d, doublet, t, triplet and m, multiplet. Electron spray ionization (ESI) mass spectra were recorded on a Finnigan, Surveyor MSQ Plus spectrometer (Thermo Finnigan, Ringoes, NJ, USA). Specific rotations of the compounds were measured at 25 °C on a Perkin-Elmer 343 polarimeter (PerkinElmer Inc., Shelton, CT, USA) using a 10 cm cell. Dichloromethane was dried by standard procedures and stored over molecular sieves. No further purification of other solvents and chemicals needed as they were reagent grade. HRMS spectra were recorded on a Bruker Maxis Impact QTOF Spectrometer (Bruker Daltonics, Billerica, MA, USA).

#### 4.1.1. Coupling Method

To a stirred solution of hydrochloride amino component (1.0 mmol) in CH_2_Cl_2_ (10 mL), Et_3_N (0.3 mL, 2.2 mmol) and subsequently WSCI∙HCl (0.21 g, 1.1 mmol) and HOBt (0.14 g, 1.0 mmol) were added at 0 °C. The acid component (1.0 mmol) was added and the reaction mixture was stirred for 1 h at 0 °C and then overnight at room temperature. After the completion of the reaction, the solvent was evaporated under reduced pressure and EtOAc (20 mL) was added. The organic layer was washed consecutively with brine, 1 N aqueous HCl, brine, 5% NaHCO_3_, and brine, dried over Na_2_SO_4_ and evaporated under reduced pressure. The residue was purified by column chromatography [EtOAc/petroleum ether (bp 40–60 °C), 2:8].

*(R)-Methyl 2-(2-(7-phenylheptanamido)hexanamido)acetate* (**6**). Yield 77%; Colorless oil; ${\left[\alpha \right]}_{D}^{20}$ + 33.0 (*c* 1.00, CH_3_OH); ^1^H–NMR (CDCl_3_, 200 MHz): *δ* 7.72 (t, *J* = 5.5 Hz, 1H, NH), 7.33–7.07 (m, 5H, arom), 6.91 (d, *J* = 8.3 Hz, 1H, NH), 4.63 (dt, *J* = 7.5 Hz, 1H, NHC*H*), 4.19–3.81 (m, 2H, NHC*H*_2_), 3.68 (s, 3H, COOCH_3_), 2.57 (t, *J* = 7.6 Hz, 2H, CH_2_), 2.19 (t, *J* = 7.4 Hz, 2H, NHCOC*H*_2_), 1.92–1.45 (m, 6H, CH_2_), 1.44–1.16 (m, 8H, CH_2_), 0.87 (t, *J* = 6.7 Hz, 3H, CH_3_); ^13^C–NMR (CDCl_3_, 50 MHz): *δ* 173.3, 172.8, 169.8, 142.4, 128.5, 128.0, 125.4, 52.6, 52.0, 40.9, 36.1, 35.7, 32.2, 31.6, 28.9, 28.8, 27.4, 25.4, 22.3, 13.8; MS (ESI) *m*/*z* (%): 391.14 (92) [M + H]^+^; Anal. Calcd for C_22_H_34_N_2_O_4_: C, 67.66; H, 8.78; N, 7.17. Found: C, 67.49; H, 8.97; N, 7.03.

*(R)-Methyl 4-(2-(2-benzamidoacetamido)acetamido)octanoate* (**10**). Yield 62%; Yellow oil; ${\left[\alpha \right]}_{D}^{20}$ + 1.5 (*c* 1.00, CHCl_3_); ^1^H–NMR (CDCl_3_, 200 MHz): *δ* 8.19–8.02 (m, 1H, N*H*), 7.87–7.67 (m, 3H, NH & arom), 7.51–7.24 (m, 3H, NH & arom), 7.03–6.86 (m, 1H, arom), 4.03 (d, *J* = 4.5 Hz, 2H, NHC*H*_2_), 3.85 (d, *J* = 5.0 Hz, 2H, NHC*H*_2_), 3.75–3.69 (m, 1H, NHC*H*), 3.53 (s, 3H, COOCH_3_), 2.26 (t, *J* = 7.3 Hz, 2H, CH_2_COO), 1.92–1.52 (m, 2H, NHCHC*H*_2_), 1.48–1.03 (m, 6H, CH_2_), 0.87–0.65 (m, 3H, CH_3_); ^13^C–NMR (CDCl_3_, 50 MHz): *δ* 174.1, 170.1, 168.8, 168.3, 132.9, 131.8, 128.4, 127.2, 51.5, 49.0, 43.9, 43.0, 34.5, 30.5, 29.7, 27.9, 22.4, 13.8; MS (ESI) *m*/*z* (%): 392.14 (90) [M + H]^+^; Anal. Calcd for C_20_H_29_N_3_O_5_: C, 61.36; H, 7.47; N, 10.73. Found: C, 61.15; H, 7.74; N, 10.54.

*(R)-Methyl 2-(2-(2-benzamidoacetamido)acetamido)hexanoate* (**13a**). Yield 53%; White solid; mp 140–142 °C; ${\left[\alpha \right]}_{D}^{20}$ + 18.6 (*c* 1.02, CHCl_3_); ^1^H–NMR (CDCl_3_, 200 MHz): *δ* 7.95–7.66 (m, 4H, NH & arom), 7.52–7.27 (m, 4H, arom), 4.45 (dt, *J* = 7.5 Hz, 1H, NHC*H*), 4.10 (d, *J* = 5.1 Hz, 2H, NHC*H*_2_), 4.06–3.82 (m, 2H, NHC*H*_2_), 3.63 (s, 3H, COOCH_3_), 1.86–1.49 (m, 2H, NHCHC*H*_2_), 1.34–1.09 (m, 4H, CH_2_), 0.80 (t, *J* = 6.6 Hz, 3H, CH_3_); ^13^C–NMR (CDCl_3_, 50 MHz): *δ* 173.0, 170.2, 169.2, 168.1, 133.2, 131.7, 128.4, 127.2, 52.4, 52.2, 43.7, 42.8, 31.4, 27.4, 22.1, 13.7; MS (ESI) *m*/*z* (%): 364.09 (90) [M + Η]^+^; Anal. Calcd for C_18_H_25_N_3_O_5_: C, 59.49; H, 6.93; N, 11.56. Found: C, 59.40; H, 7.03; N, 11.47.

*(S)-Methyl 2-(2-(2-benzamidoacetamido)acetamido)hexanoate* (**13b**). Yield 68%; White solid; mp 139–141 °C; ${\left[\alpha \right]}_{D}^{20}$ − 18.0 (*c* 1.00, CHCl_3_); ^1^H–NMR (CDCl_3_, 200 MHz): *δ* 8.04 (t, *J* = 4.9 Hz, 1H, NH), 7.88 (t, *J* = 5.1 Hz, 1H, NH), 7.83–7.69 (m, 2H, NH & arom), 7.62–7.46 (m, 1H, arom), 7.45–7.19 (m, 3H, arom), 4.40 (dt, *J* = 7.6 Hz, 1H, NHC*H*), 4.06 (d, *J* = 4.7 Hz, 2H, NHC*H*_2_), 4.00–3.80 (m, 2H, NHC*H*_2_), 3.59 (s, 3H, COOCH_3_), 1.86–1.47 (m, 2H, NHCHC*H*_2_), 1.32–1.05 (m, 4H, CH_2_), 0.77 (t, *J* = 6.7 Hz, 3H, CH_3_); ^13^C–NMR (CDCl_3_, 50 MHz): *δ* 173.0, 170.2, 169.2, 168.1, 133.1, 131.6, 128.2, 127.2, 52.3, 52.0, 43.6, 42.7, 31.3, 27.4, 22.0, 13.6; MS (ESI) *m*/*z* (%): 364.04 (90) [M + Η]^+^; Anal. Calcd for C_18_H_25_N_3_O_5_: C, 59.49; H, 6.93; N, 11.56. Found: C, 59.20; H, 7.15; N, 11.35.

*(R)-tert-Butyl 9-butyl-1,4,7,10-tetraoxo-1-phenyl-2,5,8,11-tetraazatridecan-13-oate* (**15a**). Yield 92%; White solid; mp 177–178 °C; ${\left[\alpha \right]}_{D}^{20}$ + 11.0 (*c* 1.00, CH_3_OH); ^1^H–NMR (CDCl_3_, 200 MHz): *δ* 8.25–8.07 (m, 1H, NH), 8.05–7.94 (m, 1H, NH), 7.93–7.76 (m, 3H, NH & arom), 7.75–7.58 (m, 1H, arom), 7.55–7.29 (m, 3H, arom), 4.96–4.74 (m, 1H, NHC*H*), 4.43–4.25 (m, 2H, NHC*H*_2_), 4.22–4.03 (m, 2H, NHC*H*_2_), 4.01–3.71 (m, 2H, NHC*H*_2_), 1.94–1.49 (m, 2H, CH_2_), 1.40 (s, 9H, CH_3_), 1.33–1.10 (m, 4H, CH_2_), 0.81 (t, *J* = 6.7 Hz, 3H, CH_3_); ^13^C–NMR (CDCl_3_, 50 MHz): *δ* 172.2, 169.6, 168.7, 168.5, 167.9, 133.5, 131.6, 128.3, 127.4, 81.9, 53.0, 43.5, 41.9, 33.1, 27.9, 27.5, 25.9, 22.4, 13.9; MS (ESI) *m*/*z* (%): 463.22 (90) [M + Η]^+^; Anal. Calcd for C_23_H_34_N_4_O_6_: C, 59.72; H, 7.41; N, 12.11. Found: C, 59.44; H, 7.70; N, 11.93.

#### 4.1.2. Saponification of Methyl Esters

To a stirred solution of a methyl ester (1.0 mmol) in water, 1 N aqueous NaOH (1 mL, 1.0 mmol) was added and the mixture was left under stirring overnight at room temperature. After the completion of the reaction, the mixture was washed with EtOAc (3 × 10 mL), acidified with 1 N aqueous HCl to pH 1 and extracted with EtOAc (3 × 10 mL). The combined organic layers were washed with brine, dried over Na_2_SO_4_ and evaporated under reduced pressure.

*(R)-2-(2-(7-Phenylheptanamido)hexanamido)acetic acid* (**7**). Yield 83%; White oil; ${\left[\alpha \right]}_{D}^{20}$ + 15.0 (*c* 1.00, CH_3_OH); ^1^H–NMR (CDCl_3_, 200 MHz): *δ* 9.67 (br s, 1H, COOH), 7.72–7.45 (m, 2H, NH), 7.36–6.99 (m, 5H, arom), 4.76–4.51 (m, 1H, NHC*H*), 4.13–3.86 (br s, 2H, NHC*H*_2_), 2.69–2.39 (m, 2H, CH_2_), 2.31–2.05 (m, 2H, NHCOC*H*_2_), 1.92–1.45 (m, 6H, CH_2_), 1.41–1.05 (m, 8H, CH_2_), 0.96–0.69 (m, 3H, CH_3_); ^13^C–NMR (CDCl_3_, 50 MHz): *δ* 174.3, 172.9, 172.0, 142.5, 128.2, 128.1, 125.5, 52.8, 41.2, 36.1, 35.7, 32.1, 31.2, 28.9, 28.9, 27.4, 25.5, 22.2, 13.8; HRMS (ESI) calcd for C_21_H_33_N_2_O_4_ [M + H]^+^: 377.2435. Found: 377.2438; Anal. Calcd for C_21_H_32_N_2_O_4_: C, 66.99; H, 8.57; N, 7.44. Found: C, 66.72; H, 8.86; N, 7.27.

*(R)-4-(2-(2-Benzamidoacetamido)acetamido)octanoic acid* (**11**). Yield 88%; White solid; mp 202–204 °C; ${\left[\alpha \right]}_{D}^{20}$ + 4.0 (c 1.00, CH_3_OH); ^1^H–NMR (D_2_O, 200 MHz): *δ* 7.89–7.70 (m, 2H, arom), 7.68–7.41 (m, 3H, arom), 4.07 (s, 2H, NHC*H*_2_), 3.88 (s, 2H, NHC*H*_2_), 3.82–3.64 (m, 1H, NHC*H*), 2.10 (t, *J* = 6.7 Hz, 2H, C*H*_2_COOH), 1.93–1.06 (m, 8H, CH_2_), 0.89–0.66 (m, 3H, CH_3_); ^13^C–NMR (D_2_O, 50 MHz): *δ* 182.9, 172.4, 171.4, 170.9, 132.7, 132.5, 128.9, 127.4, 50.0, 34.2, 33.6, 31.2, 27.5, 21.9, 13.4; HRMS (ESI) calcd for C_19_H_28_N_3_O_5_ [M + H]^+^: 378.2023. Found: 378.2025; Anal. Calcd for C_19_H_27_N_3_O_5_: C, 60.46; H, 7.21; N, 11.13. Found: C, 60.25; H, 7.42; N, 11.01.

*(R)-2-(2-(2-Benzamidoacetamido)acetamido)hexanoic acid* (**14a**). Yield 93%; White solid; mp 213–215 °C; ${\left[\alpha \right]}_{D}^{20}$ + 8.6 (*c* 1.00, CH_3_OH); ^1^H–NMR (DMSO, 200 MHz): *δ* 8.84 (t, *J* = 5.6 Hz, 1H, NH), 8.19 (t, *J* = 5.5 Hz, 1H, NH), 8.05–7.70 (m, 3H, NH & arom), 7.58–7.24 (m, 3H, arom), 4.22–4.02 (m, 1H, NHC*H*), 3.85 (d, *J* = 5.6 Hz, 2H, NHC*H*_2_), 3.72 (d, *J* = 5.6 Hz, 2H, NHC*H*_2_), 1.76–1.43 (m, 2H, CH_2_), 1.35–1.01 (m, 4H, CH_2_), 0.79 (t, *J* = 6.5 Hz, 3H, CH_3_); ^13^C–NMR (DMSO, 50 MHz): *δ* 173.6, 169.3, 168.8, 166.7, 133.9, 131.5, 128.3, 127.4, 51.8, 42.9, 41.8 30.8, 27.5, 21.8, 13.8; HRMS (ESI) calcd for C_17_H_24_N_3_O_5_ [M + H]^+^: 350.1710. Found: 350.1711; Anal. Calcd for C_17_H_23_N_3_O_5_: C, 58.44; H, 6.64; N, 12.03. Found: C, 58.22; H, 6.86; N, 11.87.

*(S)-2-(2-(2-Benzamidoacetamido)acetamido)hexanoic acid* (**14b**). Yield 91%; White solid; mp 192–201 °C; ${\left[\alpha \right]}_{D}^{20}$ − 8.1 (*c* 1.08, CH_3_OH); ^1^H–NMR (CDCl_3_ and drops CD_3_OD, 200 MHz): *δ* 7.82–7.58 (m, 1H, arom), 7.53–7.14 (m, 4H, arom), 4.38–4.21 (m, 1H, NHC*H*), 4.05 (s, 2H, NHC*H*_2_), 3.96 (s, 2H, NHC*H*_2_), 1.87–1.43 (m, 2H, CH_2_), 1.34–1.09 (m, 4H, CH_2_), 0.75 (t, *J* = 6.5 Hz, 3H, CH_3_); ^13^C–NMR (DMSO, 50 MHz): *δ* 173.8, 168.9, 169.0, 166.5, 134.3, 131.8, 128.7, 127.0, 51.4, 43.2, 41.1 31.2, 27.3, 21.2, 13.4; HRMS (ESI) calcd for C_17_H_24_N_3_O_5_ [M + H]^+^: 350.1710. Found: 350.1712; Anal. Calcd for C_17_H_23_N_3_O_5_: C, 58.44; H, 6.64; N, 12.03. Found: C, 58.24; H, 6.82; N, 11.90.

#### 4.1.3. Cleavage of *tert*-Butyl Protecting Group

A solution of the *tert*-butyl ester derivative (1 mmol) in 50% TFA/CH_2_Cl_2_ (0.5 M) was stirred for 2 h at room temperature. After the completion of the reaction, the organic solvent was evaporated under reduced pressure and the residue was purified by recrystallization.

*(R)-9-Butyl-1,4,7,10-tetraoxo-1-phenyl-2,5,8,11-tetraazatridecan-13-oic acid* (**16a**). Yield 87%; White solid; mp 195–206 °C; ${\left[\alpha \right]}_{D}^{20}$ + 20.0 (*c* 1.00, CH_3_OH); ^1^H–NMR (CD_3_OD, 200 MHz): *δ* 7.91–7.77 (m, 2H, arom), 7.59–7.35 (m, 3H, arom), 4.45–4.32 (m, 1H, NHC*H*), 4.26 (s, 2H, NHC*H*_2_), 4.05 (s, 2H, NHC*H*_2_), 3.90 (s, 2H, NHC*H*_2_), 1.97–1.57 (m, 2H, CH_2_), 1.41–1.16 (m, 4H, CH_2_), 0.86 (t, *J* = 6.6 Hz, 3H, CH_3_); ^13^C–NMR (CD_3_OD, 50 MHz): *δ* 173.2, 171.8, 171.2, 170.2, 169.2, 133.3, 132.2, 128.7, 127.5, 53.6, 43.7, 42.9, 31.6, 28.0, 22.4, 22.3, 13.9; HRMS (ESI) calcd for C_19_H_27_N_4_O_6_ [M + H]^+^: 407.1925. Found: 407.1927; Anal. Calcd for C_19_H_26_N_4_O_6_: C, 56.15; H, 6.45; N, 13.79. Found: C, 55.84; H, 6.62; N, 13.65.

### 4.2. Biology

#### 4.2.1. Cell Culture

The methods used for the isolation, characterization and cultivation of rat renal mesangial cells have been previously described in details [29]. Confluent cells were incubated in 24-well-plates for 4 h in serum-free DMEM including 0.1 mg/mL of fatty acid-free bovine serum albumin. Subsequently, cells were preincubated for 20 min either with the vehicle or the indicated concentrations of the various inhibitors prior to stimulation with IL1β (1 nM) plus Fk (5 μM) in the presence of the inhibitors for 24 h. The total stimulation volume per well was 500 μL. Cell supernatants were taken for PGE_2_ quantification.

#### 4.2.2. Quantification of Prostaglandin E_2_

A PGE_2_-specific enzyme-linked immunosorbent assay (ELISA) kit (from Enzo Life Science, Lörrach, Germany) was used for the quantification of PGE_2_ in the cell culture supernatants. 100 μL of the supernatants were processed according to the manufacturer’s instructions.

#### 4.2.3. Statistical Analysis

Statistical analysis of data was performed using one-way ANOVA following Bonferroni post-hoc test for multiple comparisons.
